# Study of the Photothermal Catalytic Mechanism of CO_2_ Reduction to CH_4_ by Ruthenium Nanoparticles Supported on Titanate Nanotubes

**DOI:** 10.3390/nano10112212

**Published:** 2020-11-06

**Authors:** Maria Novoa-Cid, Herme G. Baldovi

**Affiliations:** 1 Department of Chemistry, Universitat Politècnica de València, 46022 Valencia, Spain; manoci@etsii.upv.es; 2 Instituto de Tecnología Química CSIC-UPV, Universitat Politènica de Valencia, 46022 Valencia, Spain

**Keywords:** CO_2_ reduction, Sabatier reaction, photothermal catalysis, titanate nanotubes, titanates, methanation, methane, ruthenium nanoparticles, photocatalysis, solar fuels

## Abstract

The Sabatier reaction could be a key tool for the future of the renewable energy field due to the potential of this reaction to produce either fuels or to stabilize H_2_ in the form of stable chemicals. For this purpose, a new composite made of ruthenium oxide nanoparticles (NPs) deposited on titanate nanotubes (TiNTs) was tested. Titanate nanotubes are a robust semiconductor with a one-dimensional (1D) morphology that results in a high contact area making this material suitable for photocatalysis. Small ruthenium nanoparticles (1.5 nm) were deposited on TiNTs at different ratios by Na^+^-to-Ru^3+^ ion exchanges followed by calcination. These samples were tested varying light power and temperature conditions to study the reaction mechanism during catalysis. Methanation of CO_2_ catalyzed by Ru/TiNT composite exhibit photonic and thermic contributions, and their ratios vary with temperature and light intensity. The synthesized composite achieved a production rate of 12.4 mmol CH_4_·g_cat_^−1^·h^−1^ equivalent to 110.7 mmol of CH_4_·g_Ru_^−1^·h^−1^ under 150 mW/cm^2^ simulated sunlight irradiation at 210 °C. It was found that photo-response derives either from Ru nanoparticle excitation in the visible (VIS) and near-infrared (NIR) region (photothermal and plasmon excitation mechanism) or from TiNT excitation in the ultraviolet (UV) region leading to electron–hole separation and photoinduced electron transfer.

## 1. Introduction

Due to the international agreements established to decrease atmospheric CO_2_ emissions, there is a considerable interest in the capture and utilization of CO_2_ [1,2]. Among the various possible reactions using CO_2_ as reagent, hydrogenation is one of the few exothermic processes, although it requires high temperatures to overcome the kinetic barrier (300–500 °C) [3,4]. In this context, it has been recently reported that CO_2_ hydrogenation can be promoted by light using suitable photothermal catalysts [5,6]. Most of the photothermal catalysts for CO_2_ hydrogenation consist of transition metal nanoparticles (NPs) supported on high surface area solids, frequently semiconductors, and in some cases the material exhibits spatial structuring [7,8]. Among these photothermal catalysts, ruthenium NPs have been reported as active materials on different supports including black silicon as nanowires [9] or photonic crystals [10] and inert silica alloyed with gold [11]. Interestingly, the use of Ru NPs on titanium semiconductors as thermal catalyst for CO_2_ hydrogenation has been scarcely reported [12], even though titanium-containing semiconductors are the most widely used materials in photocatalysis [13]. Particularly, in the Sabatier reaction, titanate oxides are widely used instead of a typical photocatalyst such as TiO_2_ P25 due to their higher production rates working under softer reaction conditions compared to other semiconductors [14,15]. Clearly, more efforts should be made to enhance the catalytic activity of Ru NPs supported on titanium semiconductors in CO_2_ methanation, and one way is by understanding the operating reaction mechanism.

Nanotubular materials are of great interest because of their exceptional electronic and mechanical properties. Among other materials, titanate nanotubes (TiNTs) are one-dimensional (1D) semiconductors that are particularly interesting due to their robustness, high band gap and high surface area [16]. The present study reports the photothermal catalytic activity of RuO_2_ NPs deposited on Na^+^,H^+^-titanate nanotubes (Na,H/TiNTs), showing the high performance on the catalytic activity of this material. Synthesis and modification of TiNTs results in a low-cost process that could be easily scaled up. In order to understand the influence of temperature and light during catalysis, the composites were tested under different temperatures and different intensities of solar simulated light and irradiation with different regions of the light spectra.

## 2. Experimental Section

*Materials.* Aeroxide titanium oxide P25 was purchased from Evonik Industries AG, (Essen, Germany). Sodium hydroxide (98% purity) was acquired from Scharlab SL (Barcelona, Spain). Ruthenium (III) chloride hydride (99.98%) was obtained from Merck (Sigma-Aldrich, St Louis, MO, USA).

*Synthesis of Na,H/TiNT (Na,HTi_3_O_7_).* In a typical TiNT preparation, 5 g of TiO_2_ P25 were suspended in 80 mL of 10 M NaOH aqueous solution. The suspension was submitted to a hydrothermal treatment at 150 °C in a sealed Teflon autoclave for 30 h inside a Binder oven (Model ED 115, Tuttlingen, Germany). After this time, the supernatant was removed and the solid recovered by filtration and washed with deionized water. Then the solid was suspended in 80 mL of 0.1 M H_2_SO_4_ and stirred gently for 30 min at room temperature. The washed solid was collected by vacuum filtration using a 0.22 μm nylon filter and dispersed again in 80 mL of deionized water at 80 °C under slow stirring for 1 h. Afterwards, the TiNTs were filtered in vacuum and dried at 80 °C overnight for 15 h. Finally, the TiNTs were calcined for 12 h at 200 °C using a Carbolite STF 15/180 tubular furnace (Carbolite Gero, Hope, UK), equipped with a Eurotherm temperature controller (Worthing, UK) to homogenize sample crystal structure.

*Synthesis of Ru/TiNT-X samples (Ru_x_Na_y_H_z_Ti_3_O_7_).* Preconditioning of the Na,H/TiNT samples was carried out by suspending 200 mg of this solid in 200 mL of deionized water. The suspension was sonicated for 20 min and then it was stirred at 250 rpm for an additional 30 min. After this time, different amounts of RuCl_3_ (2.6, 14.5, and 46.8 mg) were dissolved in 5 mL of deionized water and added at room temperature to the preconditioned Na,H/TiNT suspensions to obtain Ru/TiNT-X samples with Ru loading of 0.5, 3.4, and 11.2 wt%, respectively. The suspension was stirred for 3 h at 70 °C. Afterwards, the grey-black solid was recovered by vacuum filtration with a 0.2 μm nylon filter and dried at 80 °C. A Na-to-Ru ion exchange efficiency of ~90% was observed in the three cases according to Na^+^ analysis of the supernatant.

*Characterization Techniques.* Diffuse reflectance ultraviolet–visible–near-infrared (UV–VIS–NIR) spectra were recorded on a Varian Cary 5000 spectrophotometer (Agilent Technologies, Santa Clara, CA, USA) having an integrating sphere and using BaSO_4_ as reference. X-ray powder diffraction (XRD) data were measured with a Bruker PANalytical Empyrean diffractometer (Malvern Instruments Limited, Malvern, UK) (Cu Kα radiation) in transmission geometry. Raman spectra of fresh Na,H/TiNTs were recorded at room temperature. In situ Raman spectra of Na,H/TiNT and Ru/TiNT-11 samples were acquired passing a gas flow of 0.05 L × min^−1^ containing 4.9 *v*/*v*% hydrogen diluted in Ar. The samples were submitted to increasing temperatures from room temperature to 150, 180, 210, and 240 °C. Spectra were recorded using a 785 nm laser excitation source on a Renishaw InVia Raman spectrometer (Wotton-under-Edge, UK) equipped with a charge-coupled device (CCD) detector. Chemical composition of the Na,H/TiNT, Ru/TiNT-0.5, and Ru/TiNT-3.5 samples was measured with inductively coupled plasma optical emission spectroscopy (ICP-OES) with Perkin Elmer Optima 2100 DV instrument (Waltham, UK) analysis to determine the metal content of the catalysts after dissolving the solids in aqua regia. For Ru-TiNT-11, this method was not suitable because ruthenium species undergo reprecipitation in the aqua regia solution. For this reason, Ru/TiNT-11 was analyzed using a scanning electron microscope utilizing a SEM, Zeiss instruments, AURIGA Compact Oberkochen, Germany) coupled with a large area Energy-Dispersive X-ray spectroscopy detector (EDS detector), X-Max 80 of Oxford Instruments (Abingdon, UK). High resolution transmission electron microscopy images (HR-TEM) were recorded on a JEOL JEM 2100F device (Akishima, Japan) with an acceleration voltage of 200 kV. In situ X-ray photoelectron spectra (XPS) of the freshly prepared and in situ reduced samples were obtained in a SPECS spectrometer (Berlin, Germany) with a 150-MCD9 detector using MgKα (hν = 1253.6 eV) X-ray radiation at an energy of 50 W. Kinetic energies of the photoelectrons were measured with a hemispherical energy analyzer (PHOIBOS 225 Mott—MCD, SPECS, Berlin, Germany) working at constant pass energy of 30 eV. XP spectra were corrected by subtraction of a Shirley background, and the spectral fitting and the peak integration were carried out using the CASA XPS software package (Version 2.3.23). XPS analysis was performed for fresh Ru/TiNT-11 and afterward the sample was submitted to 210 °C while passing a stream of 5% of H_2_ flowing at 0.05 L × min^−1^ for 30 min. After this time, the hydrogen stream was stopped, and the sample was taken to vacuum and then measured.

*Photothermal catalytic CO_2_ hydrogenation experiments.* Catalytic experiments were performed at least in duplicate, and the data points of time-conversion plots corresponded to the average of three measurements and accompanying error bar corresponded to the standard deviation. The reaction was performed under batch in a pressurized and closed reactor with a temperature-controlled system. The quartz reactor was equipped with a heating system and a thermocouple to control the reaction temperature. All reactions were performed with freshly prepared material. However, during reusability tests the catalyst sample was the same. The catalyst was activated in a pressurized reactor by heating at 200 °C with 1.2 bar of hydrogen. In each experiment, 100 mg of the Ru/TiNT-X sample were introduced inside the reactor and sealed. Initially, the reactor was purged for at least 20 min with 99.999% hydrogen flow. Then, the reactor was charged at a total pressure of 1.5 bar, corresponding to 1.2 bar of hydrogen and 0.3 bar of carbon dioxide, corresponding to 4 equivalents of H_2_ to 1 equivalent of CO_2_. The evolved gases were analyzed by injecting a small volume with a 2.5 mL Hamilton syringe (Reno, NE, USA) into an Agilent 490 Micro GC system syringe (Agilent Technologies, Santa Clara, CA, USA) charged with Molsieve 5 Å column (Agilent Technologies, Santa Clara, CA, USA), using Ar as carrier gas.

*Influence of the temperature.* Experiments were performed using a Newport solar simulator light source (Irvine, CA, USA) setting the incident light power on the sample at 100 mW/cm^2^. The reaction was carried out with the Ru/TiNT-3.5 sample and the temperatures tested were 150, 180, 210, and 240 °C. Identical experiments were repeated with the photoreactor at the same location and with the same temperature settings, but covering the photoreactor with aluminum foil ensuring that no light was transmitted to the interior of the system.

*Influence of Ru sample loading.* These experiments were performed using a Newport solar simulator light source with an incident light power of 150 mW/cm^2^. The tests were carried out placing 100 mg of Ru/TiNT-0.5, Ru/TiNT-3.5, or Ru/TiNT-11 as catalyst on the photoreactor.

*Influence of light intensity.* These experiments were performed using a Newport solar simulator light source from 100 to 1000 W setting the incident light power on the sample to 100, 150, and 230 mW/cm^2^. The temperature was fixed at 180 °C and the sample chosen for the experiment was Ru/TiNT-3.5.

*Influence of wavelength range.* The light source used was a 150 W Xe-Hg Hamamatsu lamp (Hamamatsu City, Japan). The selected sample was Ru/TiNT-11 and the reaction temperature was fixed at 210 °C. For each experiment, a different bandpass filter was utilized to select three wavelength regions, i.e., UV (280–400 nm), VIS (450–760 nm), and IR (IR ≥ 760 nm). The incident light power was adjusted to 100 mW/cm^2^ for each region.

*Reusability test.* The stability experiment was performed for Ru/TiNT-11 repeating reactions of 24 h at 210 °C and 150 mW/cm^2^ for 10 runs. Before the first use, fresh catalyst was activated with heating at 150 °C in vacuum for 15 h and, between each test, the reactor was cooled down and purged with fresh argon. Then, the photoreactor was heated at 210 °C for 6 h in the dark. After this time, the photoreactor was cooled down to room temperature and charged again with reaction gases in a proportion of 4:1 for H_2_ and CO_2_ and then heated to 210 °C to carry out the reaction for 250 min.

## 3. Results and Discussion

**Scheme 1:**
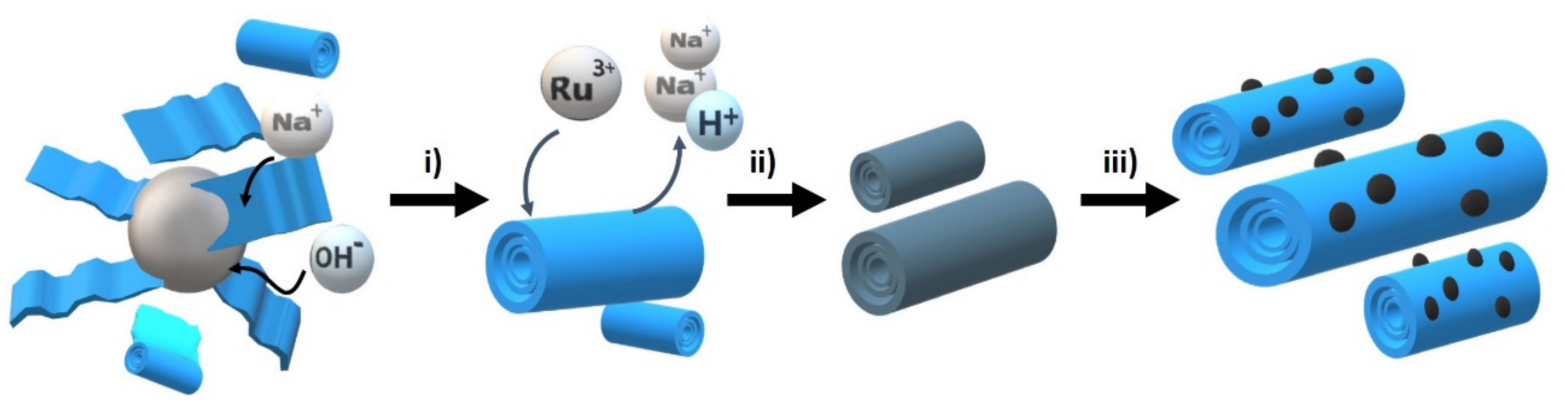
Pictorial illustration of the synthesis of ruthenium/titanate nanotubes (Ru/TiNTs) starting from TiO_2_ anatase. (**i**) Synthesis of Na,H/TiNT by hydrothermal treatment with 10 M NaOH at 150 °C for 6 h. (**ii**) Ru^3+^ to Na^+^ and H^+^ ion exchange. (**iii**) Calcination at 200 °C under air.

The materials under study were prepared starting from Na,H/TiNT through an initial ion exchange of Na^+^ by Ru^3+^ followed by thermal treatment at 200 °C under air. After this annealing, RuO_2_ NPs evolved on the surface of the TiNTs. The process is illustrated in Scheme 1. Three different samples with differences in the Ru loading were prepared by controlling the RuCl_3_ concentration in the ion exchange step (see Table 1). The difference in concentration of the RuCl_3_ used during the loading reaction had no influence on the RuO_2_ NPs’ size that remained at 1.5 nm on average (Table 1).

For Ru/TiNT-0.5 and Ru/TiNT-3.5, Ru loading was determined by ICP-EOS analysis after dissolving the solid in HF-aqua regia. Due to the precipitation of RuO_x_ species in aqua regia at high concentration of this metal in solution, the Ru content in sample Ru/TiNT-11 was determined by large-area EDX elemental analysis. The poor solubility of ruthenium species in aqua regia is a recurrent analytical issue when the material has a high concentration of this metal [17].

**Figure 1:**
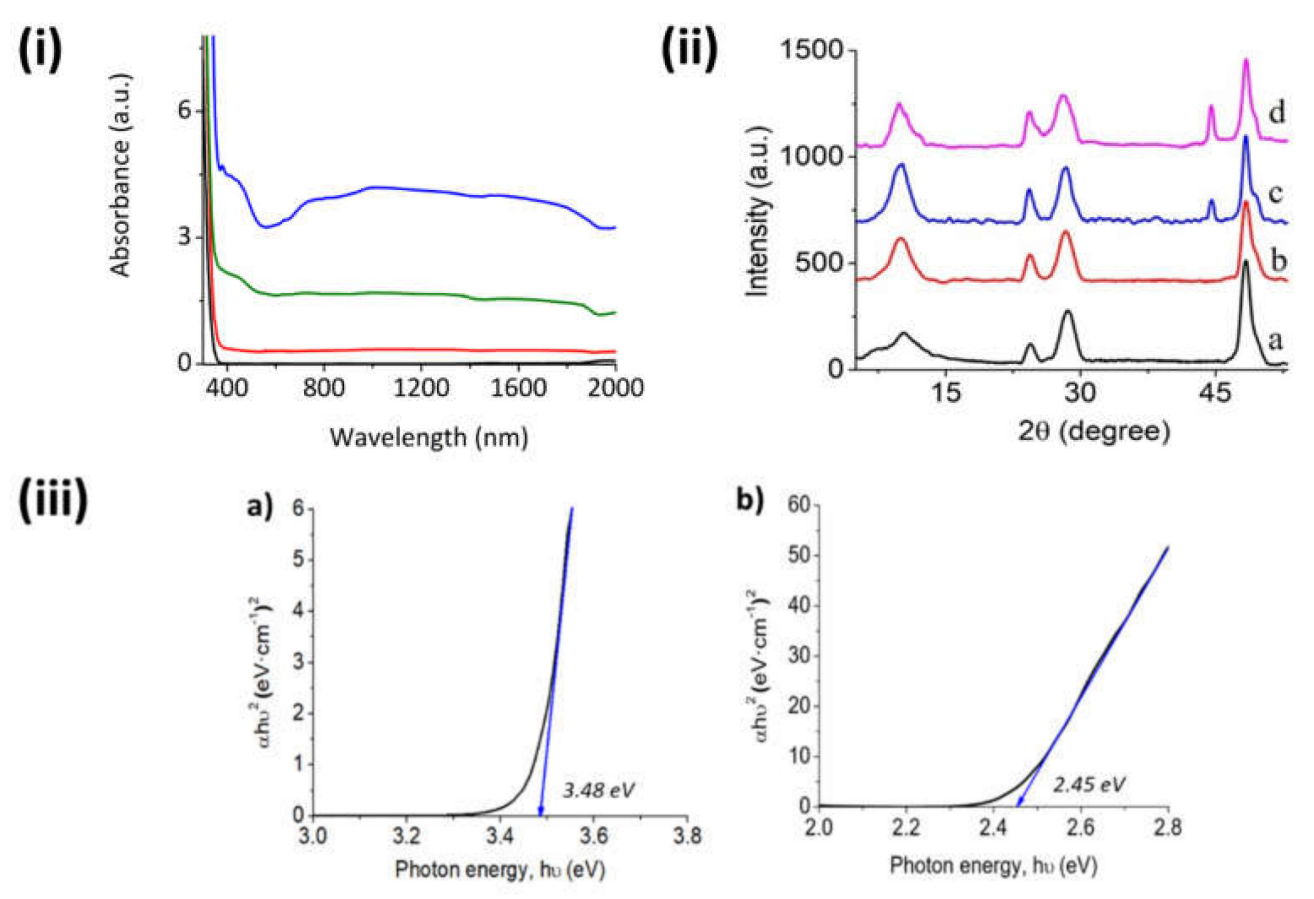
(**i**) Absorption spectra of UV–VIS–NIR acquired for Na,H/TiNT (black line), Ru/TiNT-0.5 (red line), Ru/TiNT-3.5 (green line), and Ru/TiNT-11 (blue line). (**ii**) X-ray powder diffraction spectra of (a) Na,H/TiNT, (b) Ru/TiNT-0.5, (c) Ru/TiNT-3.5, and (d) Ru/TiNT-11; RuO_2_ (rutile) (JCPDS-40-1290). (**iii**) Tauc plot assuming a direct band gap for the semiconductors (**a**) Na,H/TiNT and (**b**) Ru/TiNT-11.

Optical UV–VIS–NIR spectroscopy shows the characteristic absorption band corresponding to the TiNTs in the UV region with an onset at about 380 nm [18]. The UV–VIS–NIR spectra of the samples are presented in Figure 1i. The UV band of the TiNTs is accompanied by much weaker absorption bands in the visible (400–550 nm) and NIR (>760) regions due to the Ru NPs in the oxide form [19]. The intensity of the Ru NP bands increased with the loading of this metal and was barely detectable in the Ru/TiNT-0.5 sample due to the low loading.

In Figure 1iii are shown estimations of optical band gaps of the fresh TiNTs and the Ru-loaded sample Ru/TiNT-11. These calculations were done using the spectroscopic data from the UV–VIS–NIR diffuse reflectance data and the Tauc plot, in which an allowed direct transition model commonly used in TiO_2_-like materials was assumed. In this context, the Ru/TiNT had a lower band gap than fresh TiNTs, reaching energy values of 2.4 eV for the Ru/TiNT. This makes possible the use of photons within the three light regions, namely UV, VIS, and IR, enhancing the light harvesting of the initial TiNT.

X-ray powder diffraction (XRD) patterns cannot be attributed to any known phase of TiO_2_ as rutile or anatase. In Figure 1ii, the XRD patterns of the Ru/TiNTs have common bands with that of Na,H/TiNT, diffraction peaks appearing at 2θ values of 9.4°, 25.4°, 28.3°, and 49.0° attributable to the (020), (110), (130), and (200) faces of the TiNTs, respectively [20]. Additionally, XRD samples of the Ru/TiNT samples show for RuO_2_ only the characteristic peak at 44.8°, which means that the deposited RuO_2_ grows preferentially in the crystallographic form of the rutile with a selective crystal growth in the (120) face [21]. Although for Ru/TiNT-0.5 this peak was not detected because the TiNT is poorly loaded, these data confirm that the original RuCl_3_ that was deposited is oxidized during the synthesis process.

**Figure 2:**
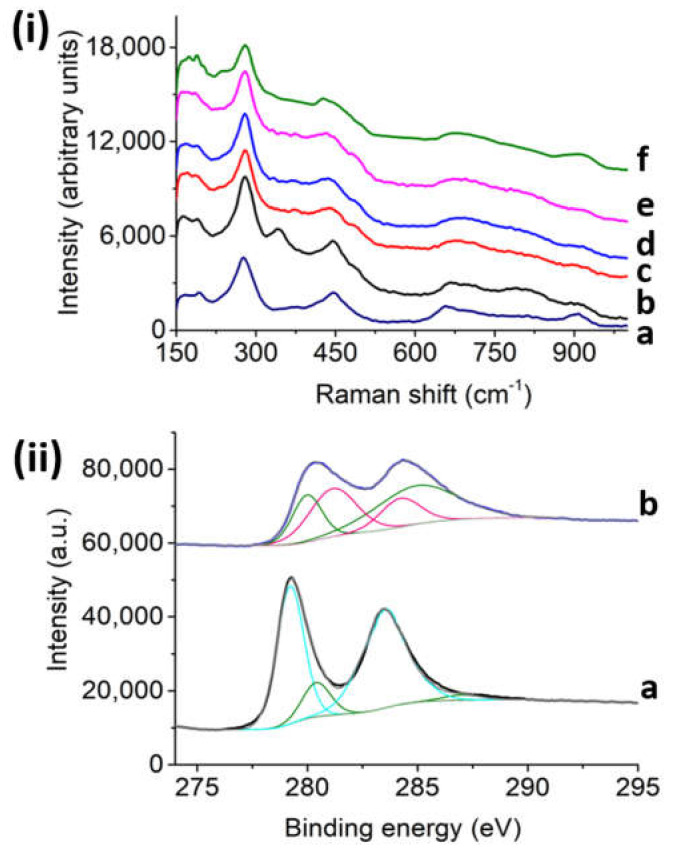
(**i**) Raman spectra recorded upon a 785 nm laser diode for the samples under study submitted to increasing temperatures from ambient to 240 °C in a chamber under hydrogen atmosphere. (a) Room temperature Na,H/TiNT, (b) room temperature Ru/TiNT-11, (c) Ru/TiNT-11 at 150 °C, (d) Ru/TiNT-11 at 180 °C, (e) Ru/TiNT-11 at 210 °C, and (f) Ru/TiNT-11 at 240 °C; all spectra have been shifted in intensity axis for clarity. (**ii**) In situ XPS spectra of sample Ru/TiNT-11: (a) recorded at room temperature under vacuum. Blue lines correspond to raw XPS spectra, pink lines denote the presence of RuO_9_ 3d_5/2-9/2_ species, and olive lines belong to the RuO_2_ 3d_5/2-9/2_ species. (b) After submitting the sample for 1 h to a 5% H_2_ stream under 210 °C, in situ XPS was recorded at 210 °C under vacuum. Black lines correspond to raw XPS spectra, olive lines belong to the RuO_2_ 3d_5/2-9/2_ species, and cyan lines refer to metallic Ru, Ru(0).

As shown in Figure 2i, the Raman spectra of fresh Na,H/TiNTs have typical peaks at 271, 445, 652, and 909 cm^−1^. On the contrary, fresh Ru/TiNT samples exhibit an intense peak at 340 cm^−1^ and some broad weak bands in the low frequency range at about 495, 705, and 802 cm^−1^; according to the literature, those peaks could be attributed to the presence of RuO_2_ in the crystallographic shape of rutile [22]. A red shift in the position of the peak corresponding to the parent Na,H/TiNT that appears at 652 cm^−1^ was observed upon RuO_2_ NP deposition, moving to 668 cm^−1^. This Raman shift suggests an interfacial interaction between both RuO_2_ and TiNT oxides. Furthermore, besides fresh samples, in situ Raman measurements of Ru/TiNTs were also carried out after 1 h exposure to hydrogen at different temperatures. The results show a complete disappearance of the 340 cm^−1^ and 705 cm^−1^ peaks, whereas the band at 495 cm^−1^ remains up to a temperature of 240 °C, at which point it also disappears. The loss of these peaks indicates that, although a great proportion of Ru atoms have been reduced to metallic form, the complete reduction of RuO_2_ does not occur at temperatures below 210 °C, explaining why the thermal catalytic activity of Ru/TiNTs increases significantly above this temperature. In situ Raman also show that while temperature increases RuO_2_ gets reduced to metallic Ru, and the TiNT peak at 448 cm^−1^ shifts to a value of 426 cm^−1^ at 240 °C. This shift suggests that oxidized and reduced Ru atoms interact with the TiNT surface in some way.

In situ X-ray photoelectron spectroscopy (XPS) was also performed to understand the catalytic mechanism by following how Ru oxidation states change. Initially, the Ru/TiNT-11 spectra were recorded at room temperature and then recorded after submitting the sample to conditions almost similar to our working conditions. For more detail, see the Experimental Section. According to the recorded XPS spectra, the Ti and Ru signals overlap in most of the energy regions; however, the oxidation states of Ru could be easily followed in the region between 260 and 300 eV [23] (see Figure 2ii). For the fresh sample, there are two peaks very close together with similar intensities that correspond to oxide species of Ru that can be assigned to the RuO_2_ and RuO_9_ species. In contrast, the heated sample under the presence of hydrogen led to a change in the spectra. This time the spectra had two peaks more separated that corresponded to Ru(0) at 279 and 284 eV that can be assigned to Ru(0) 3d5/2 and 3d9/2, respectively. However, traces of RuO_2_ were also found at the lower energies of 278 and 282.7 eV. These traces could be attributed to a small oxygen exchange after the hydrogen stream when the XPS sample chamber goes to vacuum. In our case, during catalysis, all Ru content was in the form of metallic ruthenium due to the more extreme conditions used, especially when photothermal catalysis is performed at temperatures above 210 °C.

**Figure 3:**
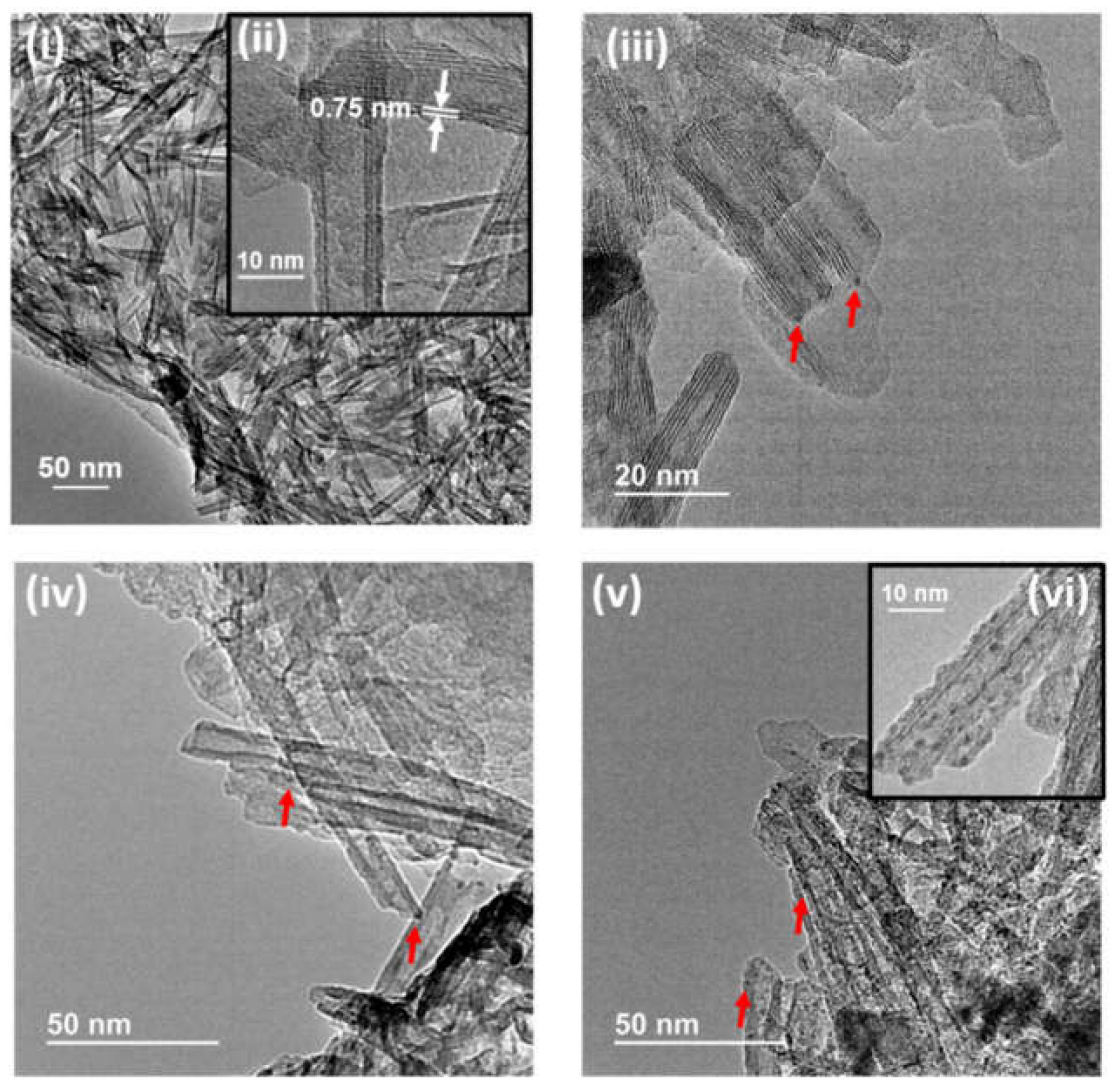
HR-TEM images of (**i**) the Na,H/TiNT; (**ii**) high-resolution image of the TiNT showing an interplanar distance of 0.75 nm; (**iii**) Ru/TiNT-0.5; (**iv**) Ru/TiNT-3.5; (**v**) Ru/TiNT-11, that comes with a high-resolution frame of a nanotube loaded with small nanoparticles (**vi**). Red arrows in the figures point to some small Ru NPs for frames (**iii**–**v**).

Morphology and size of Ru NPs were determined by high-resolution transmission electron microscopy. Representative images are presented in Figure 3. These images clearly show the tubular structure of TiNTs with an interplanar distance of 0.75 nm as show Figure 3i,ii, which corresponds to the interlayer spacing of d_200_ crystal face, typical in these materials [3]. The presence of Ru NPs is detected in all samples by the observation of small dots with dark contrast (see Figure 3iii–v). Measurements of the dimensions of a statistically relevant number of these Ru NPs show, for the fresh samples, a narrow particle size distribution of 1.4 ± 0.2 nm for Ru/TiNT-3.5 (see Figure 3vi). Surprisingly, the average particle dimension did not grow with the increase of Ru loading as can be seen in Figure 3v, and all samples had narrow size distribution. It appears that the tubular morphology or the crystal structure of Na,H/TiNTs somehow favor the growth of small NPs on their surface.

Previous reports of Walsh et al. [24] and Bavykin et al. [25] discussed the synthesis of these materials, loading different amounts of Ru, and did not find changes in the average size of the nanoparticles, although the same did not happen for other metals such as Au. They proposed that an increased ruthenium loading results in an increased density of nanoparticles deposited, rather than an increased average particle diameter. It seems that RuO_x_ NPs are highly stable regardless of the substrate used; however, agglomeration occurs after the catalytic reaction due to the formation of Ru(0), which leads to agglomeration and growth of the initial nanoparticle size. This could be explained by the absence of ionic interaction between metallic Ru and negative charges of titanate nanotubes.

### 3.1. Catalytic Activity

A series of experiments aimed at exploring the thermal and photochemical activity of the Ru/TiNT samples in CO_2_ hydrogenation were carried out.

**Figure 4:**
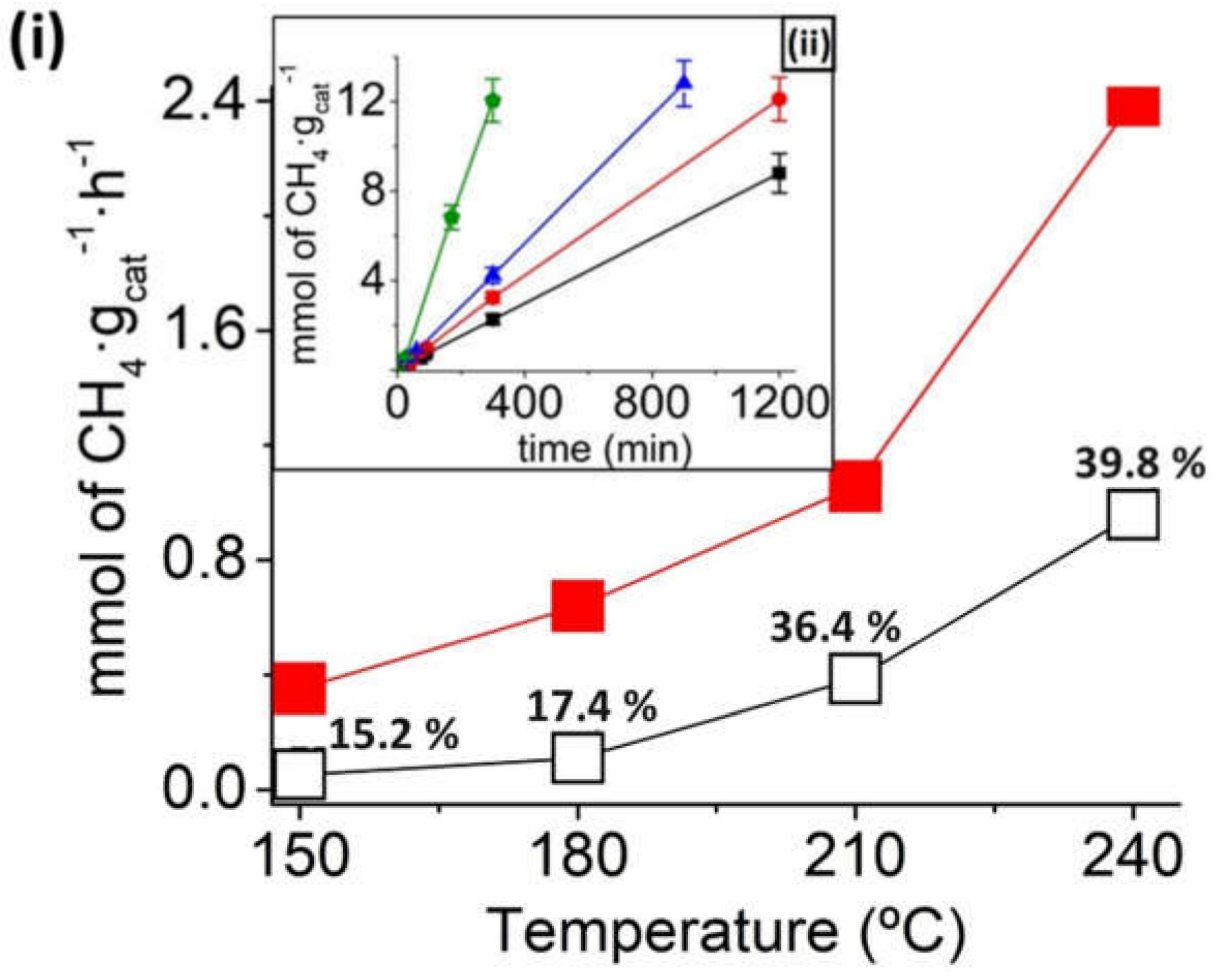
(**i**) Methane conversion rate for Ru/TiNT-3.5 as a function of the temperature for CO_2_ hydrogenation upon irradiation with 100 mW/cm^2^ light power (■) and in the dark (□). (**ii**) Methane conversion as a function of time for CO_2_ methanation under 1 sun light power (equivalent to 100 mW/cm^2^) at different temperatures (■) 150 °C, (●) 180 °C, (▲) 210 °C, and 240 °C (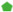).

In view of the general catalytic activity for hydrogenation, when the Ru/TiNT samples are heated to some point they can promote either in the dark or with light-selective CO_2_ hydrogenation to methane. However, traces of ethane were detected during catalysis but with small production values, lower than 1% of ethane conversion. Figure 4i shows the methane formation in the range of temperatures from 150 to 240 °C for the Ru/TiNT-3.5 sample under thermal catalysis regime and photothermal catalysis regime. Under thermal conditions, catalytic activity increases with temperature. At this point, a substantial increase in the thermal CO_2_ methanation occurs when temperatures pass 200 °C, reaching values of ~1 mmol of CH_4_·g_cat_^−1^·h^−1^ or equivalent to 28.5 mmol of CH_4_·g_Ru_^−1^·h^−1^ at 240 °C. The production rate value in mmol of CH_4_·h^−1^·g_Ru_^−1^ units was calculated with the raw production rate value CH_4_·h^−1^ and then divided by the mass of Ru deposited on the sample Ru/TiNT. As it can be seen, the amount of methane produced in the dark for temperatures below 200 °C is much less in comparison with the methane conversion under the same conditions when exposed to simulated sunlight. For example, when analogous experiments were carried out under 100 mW/cm^2^ simulated sunlight irradiation at the same temperatures, much higher rates were obtained, reaching rate values of 2.45 mmol CH_4_·g_cat_^−1^·h^−1^. The estimation of the thermal and photochemical contributions to the reaction mechanism with the production rates is shown in Figure 4ii. We can conclude that the photochemical contribution is the highest in reaction for all temperatures tested, although its importance decreases when temperature rises. For example, the methane production at 180 °C thermal contribution represents 17.4% of the methane production, whereas the thermal contribution at 240 °C represents 39.8%. Figure 4ii shows the temporal evolution of methane formation for Ru/TiNT-3.5 in the range of temperatures from 150 to 240 °C under irradiation with simulated sunlight. As it can be seen, the production rate is enhanced by 7.3 times when the photothermal catalysis was performed at 150 °C and 240 °C. However, this increase is even larger for thermal catalysis, representing an enhancement of 23 times, when the reaction is performed at the same temperatures. This fact shows that, first, the optimum working temperatures are above 200 °C and, second, the production rate of this reaction increases substantially when the catalyst is also irradiated with simulated sunlight.

**Figure 5:**
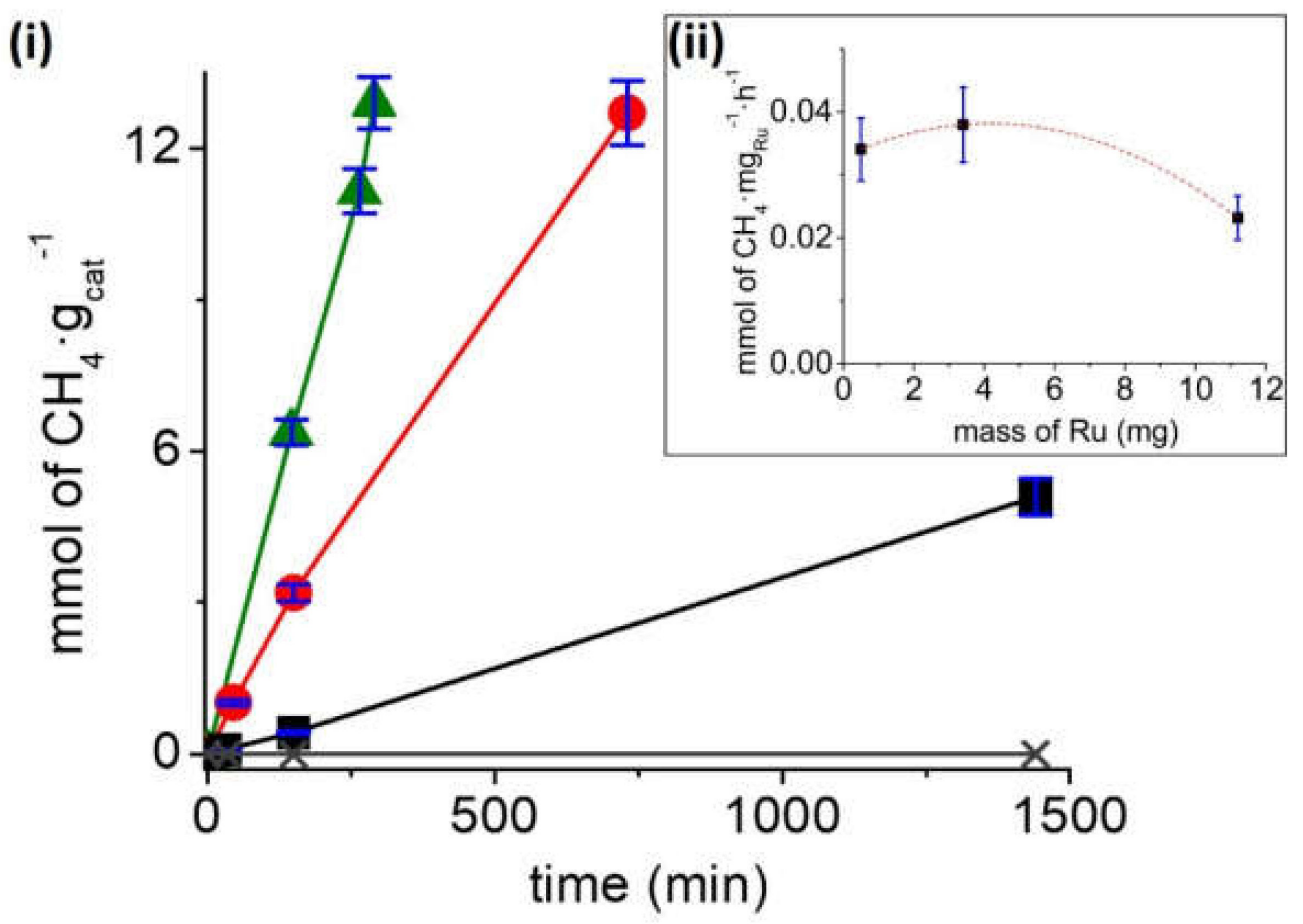
(**i**) Photothermal catalytic hydrogenation of CO_2_ to methane at 180 °C and 150 mW/cm^2^ light power in the presence of Ru/TiNT samples with different Ru content. Na,H/TiNT (**×**), Ru/TiNT-0.5 (■), Ru/TiNT-3.5 (●), and Ru/TiNT-11 (▲). (**ii**) Production rate per mg of Ru deposited on the TiNTs.

The influence of Ru loading on the TiNTs in the amounts of 0.5, 3.4, and 11.2 wt% in the photothermal catalytic activity was also studied at 180 °C and 150 mW/cm^2^ light power (see Figure 5i). At those working conditions, fresh TiNTs without Ru produce traces of methane that are negligible compared to the production of Ru/TiNT samples. In contrast, the CO_2_ methanation reaction rate increases significantly with the amount of RuO_2_ NPs deposited, measuring the highest reaction rate for the Ru/TiNT-11 sample. Furthermore, as can be seen in Figure 5ii, the catalytic efficiency per mass of loaded Ru was evaluated, indicating that the reaction rate increases almost linearly with low Ru loading. However, there is a small rate decrease when the loading is raised up to 11.2 wt%. This suggests a nonlinear relationship between the catalytic activity with the amount of mass of Ru deposited, indicating an optimal Ru loading between 3.5 and 5 wt% of Ru. As Ru NPs are the active catalyst of the reaction, a linearly increment of Ru amounts should lead to linear increments of methanation production. Nevertheless, a drop of methanation efficiency occurred, suggesting that the support piece of the catalyst must be affected. This fact is rationalized considering that the support loses the number of incident photons while it is covered by high densities of ruthenium nanoparticles (see Figure 33vi and Figure 5ii).

To determine the photothermal catalytic mechanism in the CO_2_ reduction reaction promoted by the Ru/TiNTs, two sets of experiments were carried out. In one study, the influence of light power on the methane production was measured. The second measurements determined the photo-response of the Ru/TiNT material as a function of the irradiation wavelength.

**Figure 6:**
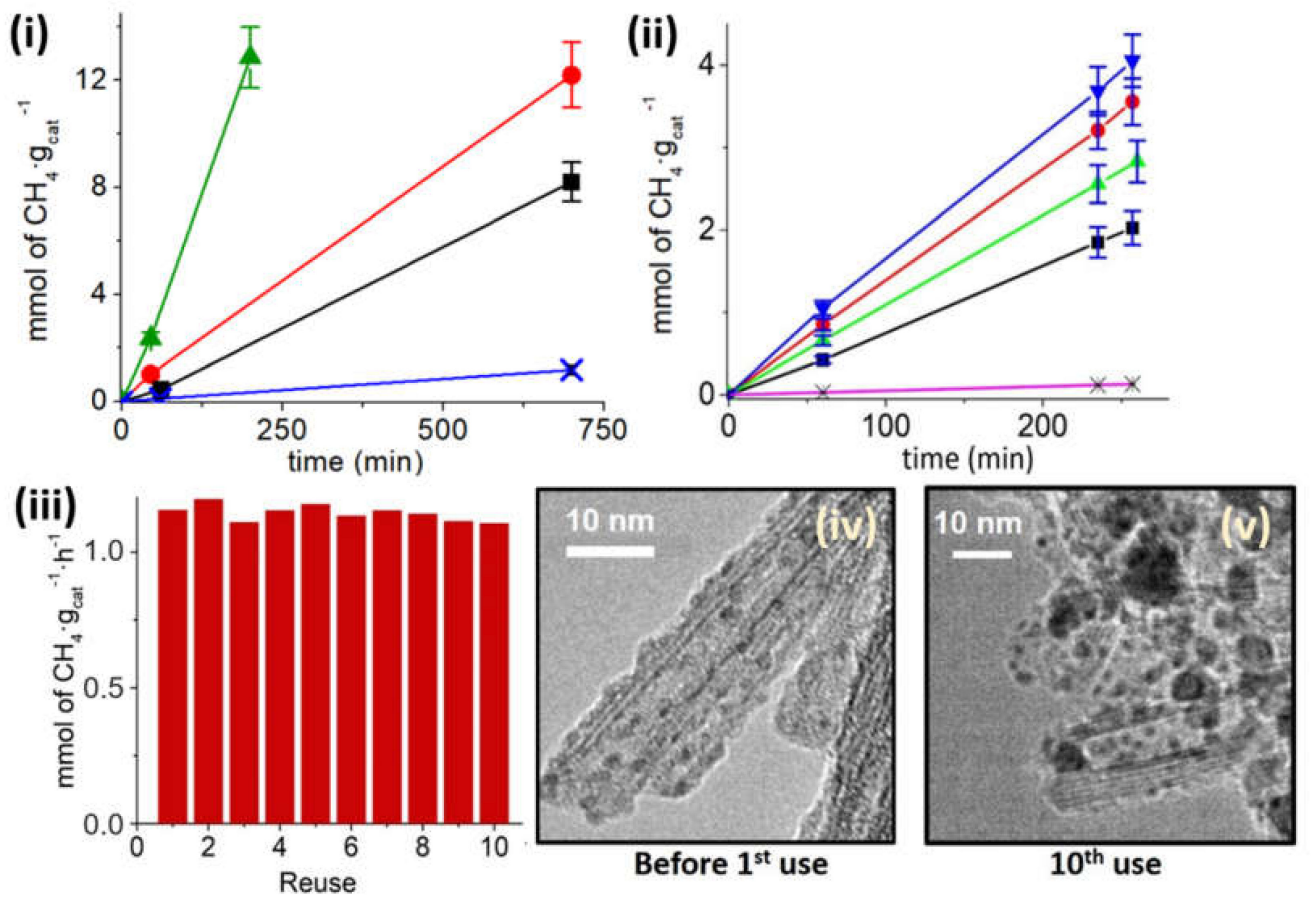
(**i**) Photothermal catalytic reduction of CO_2_ with Ru/TiNT-3.5 at 180 °C in the dark (**×**) and under irradiation with simulated sunlight of 100 mW/cm^2^ (■), 150 mW/cm^2^ (●). or 230 mW/cm^2^ (▲). (**ii**) Photothermal catalytic reduction of CO_2_ at 180 °C utilizing Ru/TiNT-11; the production was normalized according to light intensity used in each light region. Only heat (■), only simulated sunlight (×), visible (▲), near-infrared (▼), and UV (●). (**iii**) Reusability experiment for Ru/TiNT-11, performed at 210 °C upon irradiation with simulated sunlight of 150 mW/cm^2^. HR-TEM images of Ru/TiNT-11 sample: (**iv**) fresh prepared sample and (**v**) same sample after 10 cycles.

To establish the influence of the light power on CH_4_ production, a temperature of 180 °C was selected since the contribution of the thermal CH_4_ formation in the dark at this temperature is low. The light power was varied from 100 to 230 mW/cm^2^ (see Figure 6i). The results presented in Figure 4 and Table 1 show an evident increase of the methane production rate as a function of the light power. When irradiation was performed at 230 mW/cm^2^, an increase in the CH_4_ production rate by a factor of 6 with respect to the methanation rate at 100 mW/cm^2^ was measured. This behavior is in accordance with the occurrence of a photocatalytic reaction, where the reaction rate is determined by the number of photons absorbed by the system.

The photo-response of the Ru/TiNTs at different light wavelength ranges was studied by using the output beam from a 150 W xenon–mercury lamp filtered with a series of band-pass filters (see Figure 6. When productivity of methane per gram of catalyst is normalized to the light energy utilized in each region of the spectrum, it is observed that the Ru/TiNT exhibits the highest photo-response in the near-infrared region (760–2000 nm) followed by the UV zone (280–380 nm). The weakest photocatalytic activity was measured for the visible light range from 450 to 750 nm, for which photothermal catalytic CO_2_ methanation was only slightly higher than the thermal activity of the catalyst; this would be a consequence of the Ru NPs that harvest light poorly in this wavelength region. In contrast, Ru NPs have a broad band in the near-IR and can absorb these photons and transform them by internal deactivation in local heat on the nanoparticle following a photothermal mechanism. This photothermal mechanism appears to be more efficient than the classical photocatalytic mechanism involving charge carrier separation on TiNT semiconductors, which would occur upon UV photon absorption. The higher activity of Ru NPs is particularly noteworthy considering the low percentage of Ru NPs on the TiNTs and agrees with the previously commented linear relationship between CH_4_ formation rate and Ru content on Ru/TiNTs. Nevertheless, irradiation in the UV, where the TiNT component has an intense absorption band, also contributes to the photocatalytic activity of the Ru/TiNT samples, although with lesser efficiency than direct excitation to Ru NPs.

Additionally, the conversion of CO_2_ to methane was studied utilizing only simulated sunlight without applying temperature (see Figure 6ii). Although conversions were low by photocatalytic means, in the order of µmoles of methane per hour, this is further proof that titanates can interact with the Ru/RuO_2_ NPs catalyst and promote the reaction by acting as a photocatalyst.

Regarding photocatalyst stability upon reuse, Figure 6iii shows the initial methanation rate upon consecutive recycles of the same Ru/TiNT-11 sample at 210 °C under solar simulated sunlight with 150 mW/cm^2^ power. This photocatalyst and these conditions were chosen because they should be favorable for deactivation considering the highest Ru loading, elevated reaction temperature, and intense light flux. After the catalyst was activated, an almost constant initial methanation rate was observed in 10 consecutive runs. No degree of deactivation was measured, however, sintering and agglomeration were observed by microscopy analysis of the Ru NPs that grow from a narrow particle size distribution with an average 1.4 nm diameter in the fresh sample to a larger heterogeneity in the particle size with an average of 3.5 ± 0.3 nm after the 10th cycle, see Figure 6iv,v.

The catalytic reduction of CO_2_ to CH_4_ is well documented, especially for thermal catalysis using RuO_x_ NPs as a catalyst. Kwak et al. [26] describe how the selectivity of the reaction changes with temperature and they describe that, apart from CH_4_, considerable yields of CO appear when the reaction is carried out at temperatures above 300 °C. Therefore, it is not surprising that, under our catalytic experiments’ working conditions, the reduction of CO_2_ led to the selective production of CH_4_. On the contrary, during the first couple of hours of the thermal and photothermal catalysis, traces of ethane were found that were consumed throughout the 24 h of reaction. This fact suggests the possibility of producing hydrocarbons larger than CH_4_ by modifying the reaction conditions in some way.

### 3.2. Discussion of the Photothermal Catalytic Reaction Mechanism

**Scheme 2:**
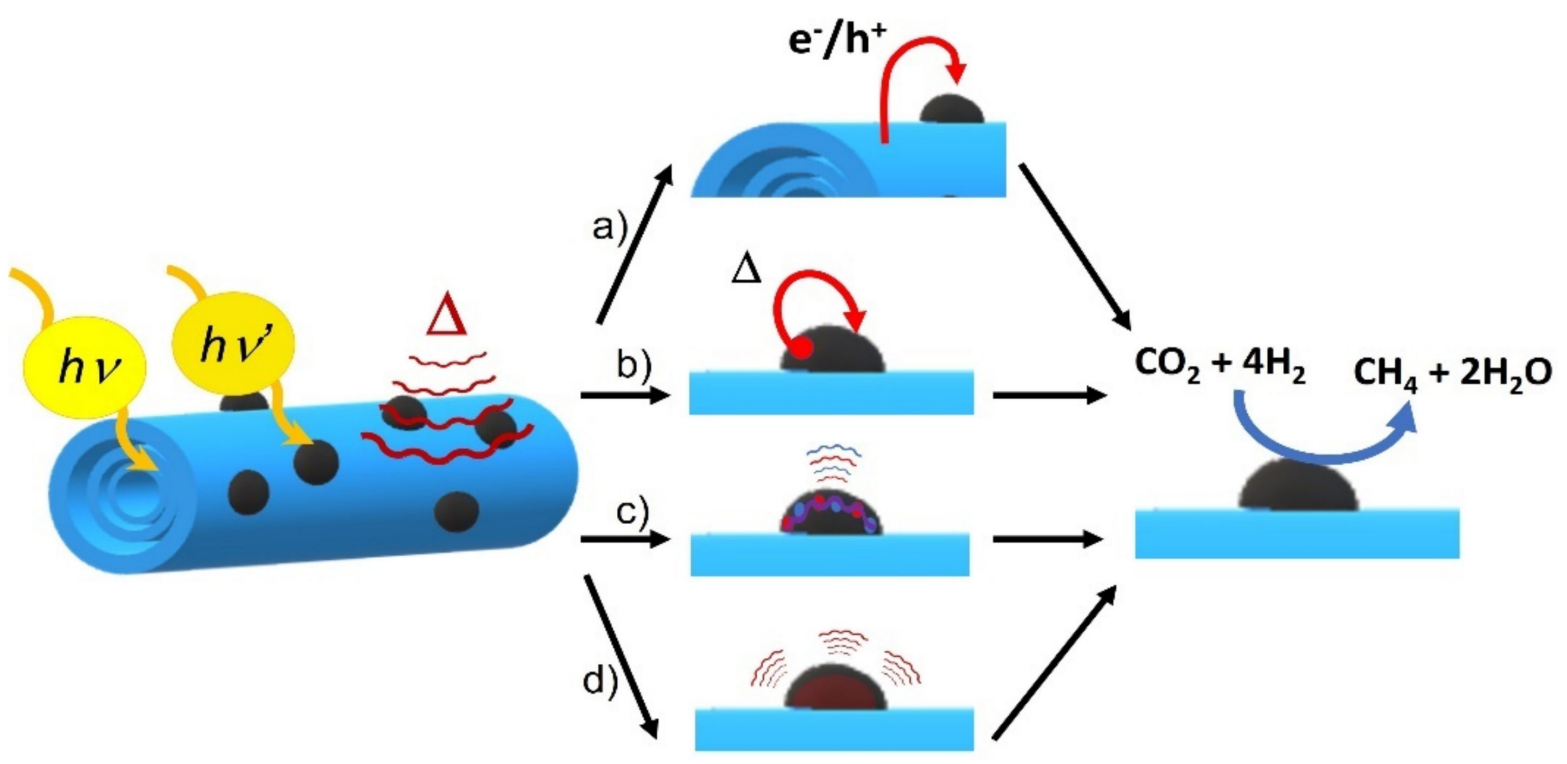
Scheme of the photothermal catalytic mechanism. Ru/TiNT composite gets irradiated and heated during photothermal reaction conditions. Photochemical contribution to catalysis: (**a**) Electron–hole charge transfer from TiNT to Ru NPs. (**b**) Excited Ru NPs deactivate by photothermal mechanism and catalyze the reaction, transferring localized heat transfer to absorbed molecules. (**c**) Plasmonic nanoparticles get excited via photon absorption and subsequently catalyze the reaction through charge transfer to molecules on the surface. Thermal contribution to catalysis: (**d**) Mechanism of thermal reaction. Ru NPs absorb heat from the environment and then transfer the heat to an absorbed reactant via heat transfer. Symbols description: hυ UV light; hυ’: VIS + IR light; Δ: heat; e^-^ electrons and h^+^ holes.

The catalytic experiments’ results show that catalysis enhances along with light power and temperature, this fact proving that CO_2_ methanation follows a dual mechanism consisting in thermal and photochemical contribution. In situ Raman measurements also show that all RuO_2_ signals disappear as temperature increases, which means that under reaction conditions RuO_2_ gets reduced to plasmonic Ru. Thermal catalysis also shows that significant methane is produced under mild temperatures (180–210 °C) compared to other publications that used dispersed Ru NPs, what means that the support enhances the Ru NPs’ activity by means of energy or heat transfer of some sort (Scheme 2d). Additionally, it was verified with Raman and XRD spectroscopy that, after catalysis, Ru NPs get reversibly oxidized to RuO_2_. Thus, the thermal reaction mechanism must be influenced by the reactivity of RuO_2_ and the Ru redox pair.

Although the TiNT was not capable of catalyzing the reaction itself under photothermal conditions, when the Ru/TiNT sample was exposed only to light catalysis did occur. Furthermore, when the composite was exposed to UV light at 180 °C, the composite was able to catalyze the reaction efficiently. In addition, when Ru loading was high, the catalytic efficiency of the composite per unit of Ru mass decreased and effectively covered the support surface, resulting in a loss of light flux hitting the support. All these facts confirm that titanate nanotubes act as a semiconductor capable of absorbing UV photons and generating electron–hole charge separation and then exchanging them with the catalyst (see Scheme 2a). In the case of Ru NPs, their photocatalytic role differs from that of TiNTs. For instance, the absorption spectra as well as the catalytic experiments show that plasmonic Ru NPs can absorb and photocatalyze the reaction utilizing visible and IR photons. In this context, photo-excited Ru NPs follow two different mechanisms to deactivate the excited state, i.e., a photothermal mechanism and a plasmonic deactivation mechanism. For the photothermal mechanism, photons are captured and then transformed by non-radiative electron relaxation into heat and transferred to near atomic nuclei that catalyze the reaction by transferring this excess of energy by heat transfer to molecules near the surface (Scheme 2b). Excited plasmonic nanoparticles could also catalyze chemical reactions through “hot electron injection”, in which the energy from surface plasmonic excited charge carriers (electrons and holes) is transferred to the molecules on the surface of the nanoparticle, as illustrated in Scheme 2c. As these two mechanisms happen in the same Ru NP, these processes might catalyze the reaction more efficiently than the photochemical catalysis of the TiNT. This fact seems to be confirmed with the experiment shown in Figure 6ii that gives higher catalytic rates upon IR irradiation. Finally, the Raman peak shifts described for the titanates when they are loaded with the RuO_2_ NPs either at room or reaction temperatures might suggest that TiNTs interact efficiently with both RuO_2_ and Ru NPs. Thus, the composite results in a synergic catalytic activity between the two components by promoting energy, heat, or charge transfer of some kind between them.

## 4. Conclusions

The tubular morphology and crystal structure of Na,H/TiNTs favor the growth of small RuO_2_ NPs on their surface that crystallize preferentially exposing the (210) face. The present study showed that RuO_2_ NPs deposited on TiNTs exhibit thermal and photocatalytic activity for the selective CO_2_ hydrogenation towards methane. Methane production increases rapidly with the reaction temperature, light intensity, and Ru loading. At temperatures below 200 °C, the thermal catalytic contribution for the Sabatier reaction is low, reaching 17.4% of the total methane conversion rate at 180 °C, but this contribution grows along with temperature. A photo-methanation rate of 12 mmol·g_cat_^−1^·h^−1^ equivalent to 110.7 mmol·g_Ru_^−1^·h^−1^ under 150 mW/cm^2^ simulated sunlight irradiation was achieved at 210 °C.

Regarding the thermal reaction mechanism, it was observed that the highest catalytic efficiency is obtained when the reaction conditions favor the situation where most of the oxidized Ru atoms are reduced metallic Ru, being the active species that catalyzes this reaction. Furthermore, the Ru/TiNT composite exhibits a dual mechanism that activates Ru for the photothermal catalytic methanation, either upon VIS and near-IR light absorption by Ru NPs following photothermal and plasmonic effect mechanisms, resulting in the conversion of the photon energy into local heat and “hot electron injection”, respectively; or upon UV light absorption by the TiNTs, leading to electron–hole charge separation and photoinduced electron transfer to Ru NPs. Ru/TiNTs maintain most of the photothermal catalytic activity upon multiple reaction cycles, although a tiny increase in the average Ru NPs was observed. Finally, the mechanical and chemical properties offered by TiNTs make them a suitable support for the future testing of new active catalysts or the introduction of metal alloys to generate other products of industrial interest.

## Figures and Tables

**Table 1:** Ru content and particle size of the Ru/TiNT samples and their corresponding photothermal catalytic activity. ^a^ Ru loading measured with ICP-EOS. ^b^ Ru loading measured with EDX. ^c^ Reaction conditions: 180 °C and 100 mW/cm^2^.

Sample	Ru wt% Synthesis	Ru wt% Deposited	RuO_2_ Particle Size (nm)	Catalytic Conversion Rate (mmol CH_4_·g_cat_^−1^ h^−1^) ^c^
Na,H/TiNT	0	0	-	traces
Ru/TiNT-0.5	0.5	0.5 ± 0.1 ^a^	1.7 ± 0.4 nm	0.17
Ru/TiNT-3.5	3.5	3.4 ± 0.1 ^a^	1.4 ± 0.3 nm	0.78
Ru/TiNT-11	11.5	11.2 ± 0.5 ^b^	1.3 ± 0.3 nm	2.91
